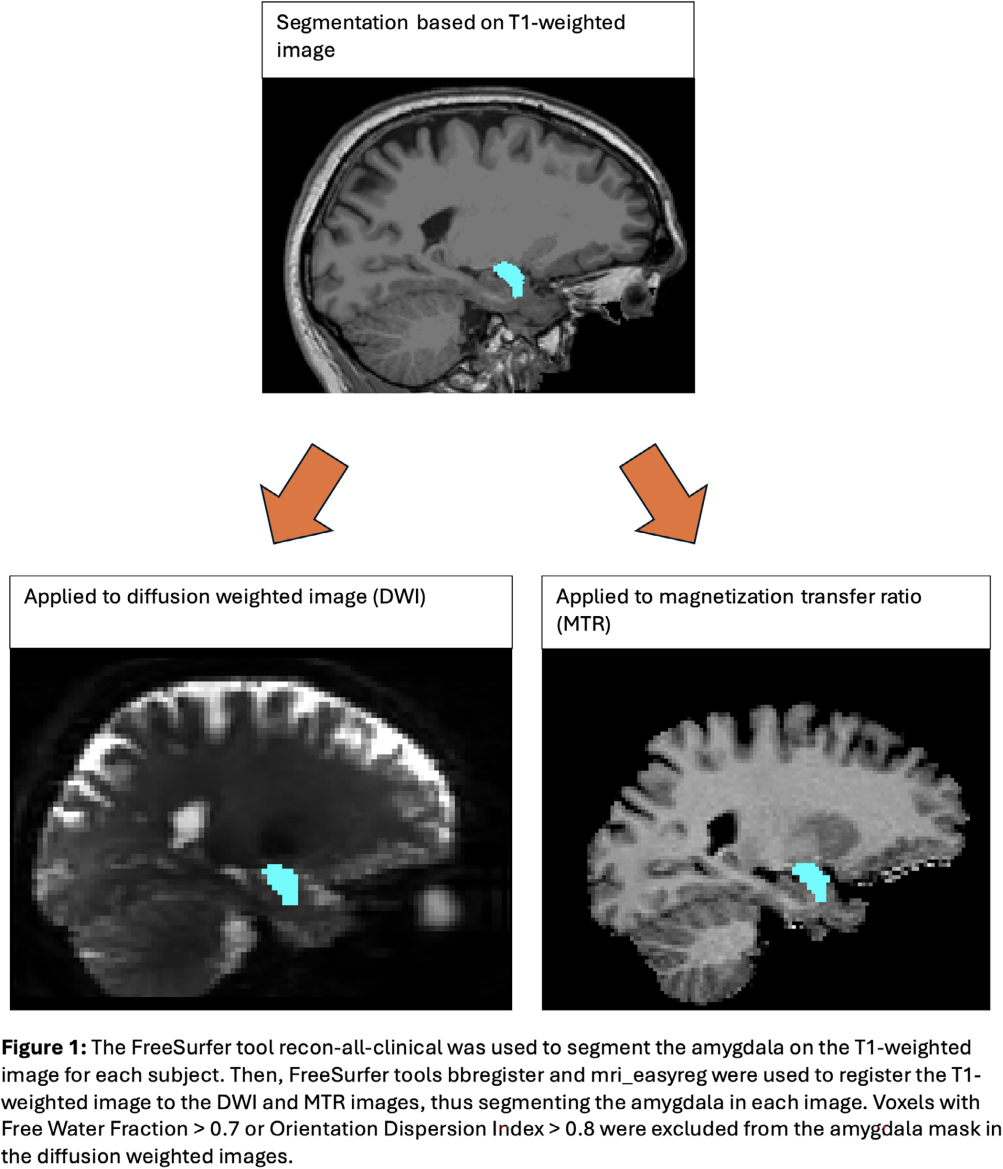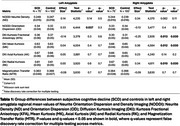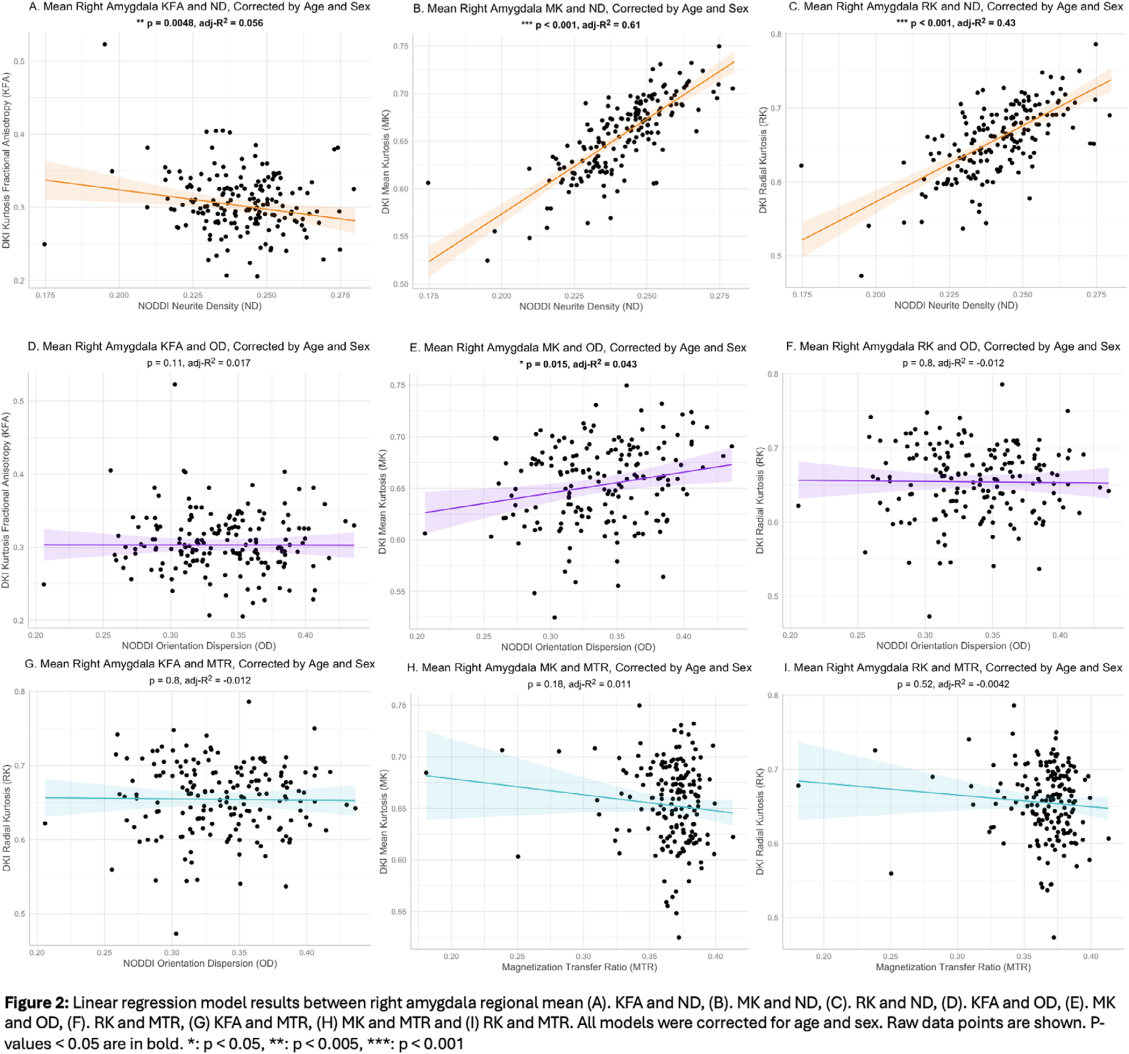# Amygdala microstructural changes in subjective cognitive decline as measured by MRI diffusion kurtosis, neurite orientation dispersion and density, and magnetization transfer imaging

**DOI:** 10.1002/alz70862_109841

**Published:** 2025-12-23

**Authors:** Ryn Flaherty, Yu Veronica Sui, Arjun V. Masurkar, Zakia Youss, Steven Baete, Thomas Wisniewski, Henry Rusinek, Mariana Lazar

**Affiliations:** ^1^ NYU Grossman School of Medicine, New York, NY USA; ^2^ Center for Cognitive Neurology, New York University Langone Health, New York, NY USA; ^3^ NYU Alzheimer's Disease Research Center, New York, NY USA; ^4^ New York University Grossman School of Medicine, New York, NY USA

## Abstract

**Background:**

Our prior work applying diffusion kurtosis imaging (DKI) to subjective cognitive decline (SCD) showed significantly decreased kurtosis fractional anisotropy (KFA) and significantly increased mean kurtosis (MK) for SCD in bilateral amygdala, an early site for tau tangle pathology^1^. However, the microstructural alterations driving these differences are unclear. Here, we assess associations of MK and KFA with neurite orientation dispersion and density imaging (NODDI) and magnetization transfer imaging (MTI) metrics, which provide a more specific characterization of tissue microstructure.

**Method:**

175 cognitively normal participants from Cam‐Can^2^ (75 SCD) ages 55‐88 were included in the analysis. Participants were defined as SCD if they endorsed problems with their memory and in the control group otherwise. Diffusion images were processed to obtain MK, Radial Kurtosis (RK), Axial Kurtosis (AK) and KFA from DKI and Neurite Density (ND) and Orientation Dispersion (OD), a marker of neurite organization, from NODDI. MTI was used to calculate the Magnetization Transfer Ratio (MTR), a marker of myelin and potentially amyloid aggregation. Mean metric values were calculated for bilateral amygdala (Figure 1). Between‐group comparisons were conducted using Wilcoxon rank‐sum tests, corrected for multiple testing. Associations between DKI, NODDI, and MTR metrics were examined using linear models corrected for age and sex.

**Result:**

SCD had lower KFA, higher MK, and higher RK in the right amygdala (Table 1). KFA had a weak negative correlation with ND, while MK and RK had strong positive correlations (Figure 2A‐C). Only MK had a weak positive correlation with OD (Figure 2D‐F). Neither KFA, MK, nor RK correlated with MTR (Figure 2G‐I).

**Conclusion:**

DKI metrics are more sensitive to amygdala changes in SCD than NODDI metrics or MTR. Lower KFA, higher MK, and higher RK were associated with higher ND but not MTR, suggestive of dendritic or glial branching. Higher MK was additionally associated with higher OD, potentially indicating reduced neurite organization. Further analyses on the impact of these amygdala changes on SCD related neuropsychiatric symptoms are needed.

1. Flaherty R, Sui YV, Li M, et al. Alzheimers Dement. 2024;20(S9):e093982.

2. Shafto MA, Tyler LK, Dixon M, et al. BMC Neurol. 2014;14(1):1‐25.